# Image captioning in Bengali language using visual attention

**DOI:** 10.1371/journal.pone.0309364

**Published:** 2025-02-13

**Authors:** Adiba Masud, Md. Biplob Hosen, Md. Habibullah, Mehrin Anannya, M. Shamim Kaiser

**Affiliations:** 1 Institute of Information Technology, Jahangirnagar University, Dhaka, Bangladesh; 2 Department of Software Engineering, Daffodil International University, Dhaka, Bangladesh; Najran University College of Computer Science and Information Systems, SAUDI ARABIA

## Abstract

Automatically generating image captions poses one of the most challenging applications within artificial intelligence due to its integration of computer vision and natural language processing algorithms. This task becomes notably more formidable when dealing with a language as intricate as Bengali and the overall scarcity of Bengali-captioned image databases. In this investigation, a meticulously human-annotated dataset of Bengali captions has been curated specifically for the encompassing collection of pictures. Simultaneously, an innovative end-to-end architecture has been introduced to craft pertinent image descriptions in the Bengali language, leveraging an attention-driven decoder. Initially, the amalgamation of images’ spatial and temporal attributes is facilitated by Gated Recurrent Units, constituting the input features. These features are subsequently fed into the attention layer alongside embedded caption features. The attention mechanism scrutinizes the interrelation between visual and linguistic representations, encompassing both categories of representations. Later, a comprehensive recursive unit comprising two layers employs the amalgamated attention traits to construct coherent sentences. Utilizing our furnished dataset, this model undergoes training, culminating in achievements of a 43% BLEU-4 score, a 39% METEOR score, and a 47% ROUGE score. Compared to all preceding endeavors in Bengali image captioning, these outcomes signify the pinnacle of current attainable standards.

## 1 Introduction

Although artificial intelligence, computer vision, and natural language processing have significantly advanced image captioning, more thorough research is still needed to address the challenges of producing accurate and contextually relevant image captions in languages with complex linguistic structures, such as Bengali. Research on image captioning and attention processes has advanced. Still, there needs to be more investigation into the specific difficulties of languages like Bengali, including linguistic complexities and the absence of well-curated captioned image libraries. This lack of knowledge demands the creation of unique end-to-end architectures that successfully capture the interdependence of visual and verbal representations within the context of Bengali while also accommodating the spatial and temporal properties of images [1]. Furthermore, given that the present criteria may not effectively reflect the caliber of generated captions, the performance evaluation measures must be improved or broadened to include Bengali’s unique linguistic peculiarities. Closing this research gap will open the door to a more thorough and efficient method for automatically creating picture captions in linguistically challenging languages, helping to depict the varied linguistic landscape of the globe more inclusively and accurately.

Researchers have explored various techniques using deep learning, achieving promising results in languages like German, Chinese, Arabic, and even Bengali [2–8]. However, Bengali image captioning still needs to be explored compared to other languages. This research addresses this gap by considering deep learning methods for Bengali image captioning. Our approach leverages existing resources like the “Flickr 8k dataset,” but with Bengali captions. This study employs the power of deep learning to generate accurate and grammatically correct captions in Bengali. The key contributions of this research are:

- Creating a dataset valuable for future research and development in Bengali image captioning models.
- Proposing a Bengali captioning model that focuses attention on features extracted by two separate Convolutional Neural Networks (CNNs).
- Conducting an extensive analysis of the proposed model, including performance comparisons with existing models, to evaluate its effectiveness in generating accurate and natural Bengali captions.
- Performing SHAP analysis to identify the most significant regions of the image contributing to image captioning.

The rest of the paper is organized as follows: Section 2 reviews related work. Section 3 provides a detailed explanation of the structure and design of the system model. Section 4 illustrates the implementation of the model. Section 5 presents a quantitative analysis. Section 6 comprehensively summarizes the paper’s findings and their implications for further research.

## 2 Related works

The research gap here lies in the need for more attention given to the Bengali language’s unique linguistic characteristics and challenges. Attention-based hybrid deep learning approaches can address Bengali linguistic nuances. Zaoad et al. [9] proposed an attention-based hybrid deep learning approach where the encoder converts the image features into feature vectors, and an RNN is utilized to construct the linguistic description. They compared their proposed model with other existing methods for Bengali image captioning. However, their study needs a more comprehensive comparative analysis, considering various state-of-the-art models and evaluating performance metrics across dimensions such as accuracy, speed, and robustness.

Solomon et al. [10] identified a notable research gap in the limited exploration of attention-based deep neural networks for image captioning in the context of the Amharic language. According to them, existing literature predominantly focuses on widely spoken languages, leaving a shortage of comprehensive investigations into the unique linguistic nuances of Amharic. Furthermore, more hybridized approaches must be tailored specifically for generating image captions in Amharic that effectively leverage attention mechanisms and deep neural networks. Bridging this gap is crucial for enhancing the accuracy and linguistic appropriateness of generated captions in Amharic, paving the way for more effective and culturally relevant applications of image captioning technology in the Amharic-speaking community. Although the conventional CNN-RNN method has been successful, it can be improved by redefining attention processes for more precise visual-linguistic alignment.

El-Gayar [11] proposed an automatic image captioning system that explicitly leverages semantic relations through deep visual attention prediction. Existing image captioning models often focus on generating captions based solely on visual content but may need a systematic approach to incorporating semantic relationships within the image. The novelty of this research lies in exploring and exploiting deep visual attention mechanisms to enhance the generation of captions that not only describe visual elements but also capture meaningful semantic connections. This approach addresses a crucial gap in the field, aiming to produce more contextually rich and semantically accurate image captions by integrating deep learning techniques with a focus on semantic relationships. Unlike high-density text categorization, which generalizes detection and recognition with single-sentence characterizations, image captioning generates descriptions for entire images. Additionally, both generation and information extraction settings show speed and accuracy advantages over baseline methods based on the state-of-the-art approach.

Han et al. [12] focused on image descriptions based on structural words, utilizing deep machine translation to create semantic representations and descriptions through a multi-tasking approach. Their method employs an LSTM-based digital signal processing model trained to transmit and receive learning strategies pertinent to this objective. This approach emphasizes the integration of structural word patterns with advanced deep-learning techniques to enhance the accuracy and richness of image descriptions.

Raza et al. [13] included a statement image and object frame collection, a new statement image description and search benchmark collection, and 8,000 images with five different captions in their study. Their review thoroughly analyzes each model’s architecture, training methodology, and performance metrics.

Yang et al. [14] reported that AIPs-SnTCN, leveraging attention mechanisms and temporal convolutional networks, demonstrates superior temporal modeling and prediction accuracy performance. Additionally, pAtbP-EnC, which combines attention-based transformers with ensemble learning, showcases remarkable results in enhancing prediction robustness and generalization across diverse datasets.

According to Akbar et al. [15], Deepstacked-AVPs, a variant of stacked autoencoders, exhibit promising results in feature extraction and dimensionality reduction, leading to improved prediction accuracy. Meanwhile, cACP-DeepGram employs deep convolutional architectures with attention mechanisms to achieve state-of-the-art results in capturing complex patterns. Finally, iHBP-DeepPSSM integrates deep learning with position-specific scoring matrices, offering enhanced prediction capabilities by incorporating evolutionary information and structural context [16]. These models collectively represent significant progress in structure prediction, addressing various challenges.

There is an increasing demand for image captioning systems that can operate seamlessly in resource-constrained environments, such as mobile devices or edge computing platforms. Current deep learning models are computationally intensive, which limits their applicability in real-time scenarios. Chandra et al. [17] aim to bridge this gap by proposing models that excel in generating accurate image descriptions while also prioritizing efficiency. Their research contributes to developing a more practical and widely applicable model by addressing computational efficiency in image captioning. This ensures that advancements in deep learning are accurate and accessible with limited computational resources.

Wajid et al. [18] highlighted a research gap related to the lack of standardized datasets and evaluation metrics specifically tailored for assessing the performance of deep learning and knowledge graph-based approaches in image captioning. Their study addresses this gap by critically evaluating existing datasets and metrics, offering insights into their limitations, and proposing recommendations for establishing benchmarks that align with the unique challenges posed by knowledge-enriched captioning systems. Additionally, the research gap involves limited attention to human-centric evaluation metrics for assessing the quality of generated image captions. While automatic metrics are commonly used, exploring and prioritizing metrics that align more closely with human perceptual judgments is necessary, ensuring that captions generated by deep learning models are accurate, contextually relevant, and coherent.

Xu et al. [19] identified a gap in the literature related to the limited exploration of deep image captioning models’ ability to generalize across diverse domains. Many existing methods are tailored to specific datasets or domains, which limits their generalizability. Their research aims to address this issue by highlighting the challenges and proposing potential solutions for achieving robust performance across various image categories and contexts.

The fusion of global and local image features for content description, particularly in the context of the Chinese language, has garnered significant attention in recent literature. Kong et al. [20] have explored various approaches to combine global features, which capture overall image characteristics, with local features that focus on specific regions or objects within an image. This fusion strategy aims to enhance the accuracy and comprehensiveness of image content descriptions, especially in complex scenes or with diverse visual elements. Additionally, integrating linguistic and semantic analysis techniques tailored to the Chinese language has been a focal point, given the unique characteristics and complexities of Chinese characters and semantics. Research in this area has shown promising results in generating detailed and contextually relevant descriptions of images in Chinese, facilitating applications in image retrieval, automatic captioning, and visual content analysis. However, challenges remain in optimizing fusion algorithms, handling variations in image content and context, and ensuring the accuracy and coherence of generated descriptions. Further exploration of multimodal fusion techniques, refinement of language modeling approaches specific to Chinese semantics, and the development of benchmark datasets and evaluation metrics tailored to image content description in the Chinese language are required.

Top-level critics and several co-critic infrastructures are used to guide the captioner. Chen et al. [21] proposed a critic-based planning technique for selecting improved captions without requiring constant supplementary monitoring (e.g., tags). In [22], major image groups are represented by a generic block diagram and a classification scheme for image captioning techniques. Measurement matrices and data points related to these techniques have also been discussed and accomplished through deep learning methods.

Sow et al. [23] utilized deep learning to generate automatic descriptions from natural images, integrating object recognition and computational linguistics. Their approach uses a convolutional layer as an image encoder, and a recurrent neural network (RNN) serves as the decoder. This setup represents the bidirectional relationship between images and their descriptive captions. In real time, the RNN generates descriptions that reflect the current state of the image.

Das et al. [24] addressed a notable gap in the literature concerning the need for studies focusing on sentiment analysis within the Assamese language. Most existing sentiment analysis frameworks predominantly cater to widely spoken languages, leaving a research void in understanding and interpreting sentiment in Assamese text, especially when combined with visual information. This research gap extends to the under-explored use of late fusion strategies for integrating image and text modalities in Assamese sentiment analysis. The study aims to address this gap by proposing and evaluating the effectiveness of late fusion techniques, providing insights into how combining textual and visual features can enhance sentiment analysis in Assamese news articles.

Badhe et al. [25] highlighted a gap in the current literature regarding remote sensing image captioning. While advancements have been made in caption generation for general images, there needs to be dedicated exploration and methodologies specifically designed to address the unique challenges posed by remote sensing imagery, such as complex geographical features and specialized content. They also identified a research gap related to the need for more incorporation of attention mechanisms in existing models for remote sensing image captioning. Attention mechanisms are crucial for focusing on relevant regions of an image. The proposed research aims to investigate the impact of these mechanisms on generating accurate and contextually meaningful captions for remote sensing imagery.

Patra et al. [26] identified a gap in the literature concerning the need for dedicated systems and methodologies for scene text editing tailored explicitly to the Bengali language. While advancements have been made in text editing for widely spoken languages, research is needed to address the unique linguistic characteristics of Bengali script and the context of scene text.

There are numerous embedding techniques available. To enhance the precision of retrieval-based image description, Ishmam et al. [27] introduced a technique called Stacked Auxiliary Embedding, which transfers information from annotated images. Anderson et al. [28] proposed approaches focusing on top-down and bottom-up attention mechanisms. The top-down approach uses Faster R-CNN to assign feature vectors to image regions, while the bottom-up approach involves establishing feature weightings. This results in the emphasis on various aspects of the image, whether small or large, leading to several overlapping sections of different sizes and proportions, each centered on distinct objects, clusters of objects, or other visually distinct regions of the image.

Mazumdar et al. [29] compared the k-nearest neighbor model, multimodal RNN, and detector-conditioned models in their study. They investigated language anomalies, repeated captions, and overlapping datasets. To generate tokens for tasks like machine translation and image captioning, RNNs need to be trained effectively. In their approach, a token produced by the model replaces the previously unknown token during the training phase. This method allows a smooth transition from an entirely directed scheme based on the actual preceding token to a less guided scheme based on the generated token. The residual networks generated by this approach are more accessible to tune than deeper neural networks, although deeper residual networks can achieve more accurate results.

These related works have significantly influenced the current study by emphasizing the importance of attention mechanisms, semantic relationships, and efficient models for image captioning. They provide valuable insights into addressing linguistic nuances, computational efficiency, standardized datasets, and evaluation metrics. Building upon these findings, this study aims to develop a more robust and contextually relevant image captioning system that can operate efficiently across diverse environments and languages.

## 3 System model

**Fig 1:**
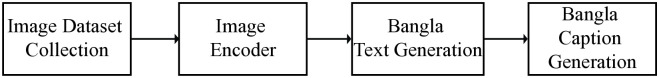
Block diagrammatic representation of image caption generation.

**Fig 2:**
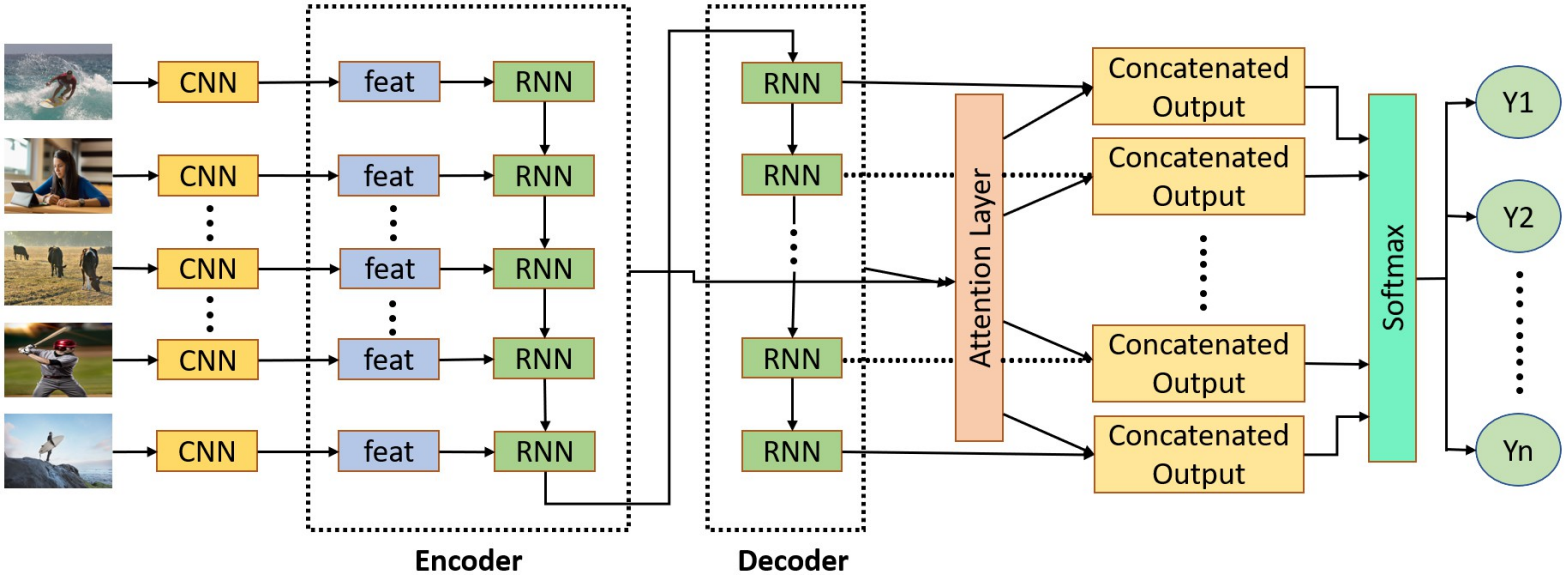
Image captioning using encoder-decoder LSTM.

This research focuses on short-form image captioning in Bengali, aiming to develop a system that generates textual descriptions from images. Fig 1 illustrates the overall image caption generation process. It begins with the data collection process, followed by the presentation of word embedding, feature extraction, and the encoder and decoder techniques. The study then introduces an attention-based decoder technique, which focuses on specific regions of the encoder’s outputs. This technique combines weights learned from the encoder with caption embeddings from the embedding layer. Both the input and output are sequences: images serve as input, and strings of descriptive words serve as output. To achieve our goals, we employ an LSTM-based many-to-many sequence model. Unlike many RNNs, this model includes internal gates that facilitate efficient back-propagation across time, addressing potential vanishing gradient issues. It consists of an LSTM encoder and an LSTM decoder. The encoder processes data from image frames, while the decoder generates textual descriptions, as shown in Fig 2.

### 3.1 Data collection

This study utilized publicly available Flickr 8k Dataset (https://www.kaggle.com/datasets/adityajn105/flickr8k), a well-established benchmark for sentence-based image description and search tasks. This dataset consists of 8,000 images, each accompanied by five captions that describe the key entities and events within the image. The original English captions are translated into Bengali, considering the limitations of machine translation tools like Google Translate. Following this, we meticulously address grammatical errors to ensure the captions reflect natural Bengali fluency.

### 3.2 Feature extraction

Each image is divided into eighty frames of varying durations and fed into a pre-trained CNN model to create a feature vector. The dimensions of each frame are reduced to 224x224 pixels before input into the CNN model using the RGB standard color space. Consequently, a tensor with dimensions 224x224x3 is input into the CNN model. Following the processing of each frame, the model outputs a vector containing 4096 values. This study compares three distinct CNN models (VGG19, ResNet-50 v2, and Inception v3) with necessary RNN models (GRU, LSTM, and BiLSTM) to determine which CNN-RNN coupling produces the best results. These CNN models significantly contribute to feature extraction due to the extensive research and development involved. The VGG19 model effectively extracts essential data using a kernel size as small as 3x3, where 19 refers to the model’s depth. This model utilizes 4096 variables for each image frame to represent the content. In contrast, deep neural networks trained using a skip connection strategy, such as those presented by Residual Networks (ResNet), can circumvent the issue of vanishing gradients entirely. These networks can contain up to 100 layers. The Inception model uses scaled filters to handle images where the primary object may have multiple representations. Additionally, the output dimension of the latter two CNNs is a vector with 2048 dimensions, indicating that each CNN uses 2048 variables to represent a frame. A convolutional neural network processes each image by performing a 2D convolution over each of the image’s three color channels. Feature vectors of varying dimensions are extracted from the input images. Both pre-trained versions of InceptionV3 and Xception are employed as CNNs [30]. To train these architectures, a very large number of images are required. The pre-trained models on ImageNet are used, but the last SoftMax layer is skipped due to insufficient images. Instead, each topology uses the previous convolutional layer for feature extraction. Each image is scaled down to 299x299 pixels before the feature extraction process.

### 3.3 Word embedding

To ensure that terms with the same meaning have consistent spelling, each word in a dictionary is represented by a vector of integers. The tokenizer class provided by Keras is used to generate a vocabulary of 1,500 words. This vocabulary is constructed using real-valued vectors, each representing an individual entry. The word2vec technique and the Keras tokenizer class are employed to generate this vocabulary, which accounts for homonyms. Each word in the training set is assigned a possible starting and ending sentence, and the validity of these sentence pairings is evaluated. The study focuses on captions that are six to ten words long, excluding whole phrases. The selected sentences help to identify the 1,500 most frequently occurring words, which are then added to the lexicon, serving as an index of keywords. Sentences are represented by integer index values, followed by padding if necessary. During the training phase, captions (words) are input in this manner and sent to the LSTM encoder. The same vectorized data is used for forward and reverse mapping to generate the appropriate Bengali textual caption.

### 3.4 Encoder and decoder

The encoder is a pivotal element in a recurrent neural network (RNN) model and is responsible for inputting the image frame properties. Given that RNN models in this study function as many-to-many sequence models, implying inconsistent lengths between input and output sequences, the encoder’s role is crucial in converting input data into a suitable intermediate form. The decoder then processes this representation to generate an appropriate output sequence, which may differ in length from the input. The final state of the encoder, serving as the intermediate representation, is passed to the decoder as its initial state, given its significance as the encoder’s endpoint. The encoder design comprises eighty cells, each corresponding to one of the eighty image frames. In this research, a total of 512 hidden layers of encoders are employed. Consequently, through an iterative process, encoder cells transform the characteristics of each image frame into a 512x512 pixel vector. Encoder cells in RNNs may vary in architectural patterns, depending on the RNN type. Notably, LSTM cells differ from GRU cells in that LSTM cells contain both cell state and hidden state, while GRU cells only include a hidden state. This distinction arises from LSTM cells’ ability to store multiple states, owing to their larger size. BiLSTM, resembling LSTM internally, incorporates an additional LSTM layer in each encoder cell.

The decoder at the opposite end of the RNN model is crucial in generating predictions for an image’s caption. It utilizes the intermediate representation of the encoder, known as the encoder’s output vector. However, to enable the decoder cells to learn the mapping function effectively, they require both the encoder output vector and the vector representation of reference captions. This process is essential for comprehending the mapping function. The “bos” token, denoting the “beginning of a sentence,” is taken by the first decoder cell to anticipate the initial vector, initiating the procedure. Similar to how the output of one decoder cell is utilized by the next to predict subsequent vectors, the process continues until an end-of-sentence (eos) token is encountered. The decoder model consists of 512 hidden states, resulting in 512x512-bit vectors for each decoder output (with a maximum caption length of 10). The decoder’s output is then passed to a dense layer with a spatial dimension of 1500 for the output space.

### 3.5 Attention mechanism

The most significant drawback of a design based on recurrent neural networks (RNNs) is volatility. If the encoder generates an inaccurate summary, the output of the language model will also be affected. Additionally, due to the vanishing gradient issue, RNNs need help remembering long sequences. When constructing longer sentences, the encoder often fails due to this constraint, known as the long-range dependency problem for RNNs and LSTMs. To mitigate this issue, the attention mechanism is crucial. It enables the neural network to select specific features by focusing on a subset of the inputs it receives. Consequently, whenever the model generates a phrase, it searches the encoder’s hidden states for a group of points containing the most relevant information. RNNs are commonly used in tasks such as generating word-for-word image captions and predicting current phrases based on visual content. However, the current word prediction often includes irrelevant visual content, complicating the creation of an appropriate caption. An attention mechanism that incorporates spatial and temporal information for image captioning is proposed to address these issues. By doing so, the decoder can selectively use the most pertinent data from the input sequence. This is achieved by combining the outputs of all the encoders into a weighted sum, giving higher weight to vectors that are more relevant to the task. Utilizing this attention strategy, the study compares the effectiveness of GRU, LSTM, and BiLSTM models.

## 4 Implementation of system model

Google Collaboratory is used extensively throughout this study as a testing ground. This Python development environment includes essential deep-learning tools like NumPy, TensorFlow, and Keras. In addition, a local computer is typically used to prepare datasets and extract image features. Spyder, an IDE, is the client machines’ default Python development environment.

### 4.1 Hyperparameter selection

Hyperparameters, which necessitate external adjustments, are pivotal factors in defining a model’s performance. Various crucial hyperparameters are carefully considered to determine the configuration that delivers optimal results for a specific model. Permutations of all the hyperparameters are shown in Table 1, where they are evaluated to determine which combination produces the highest-quality output.

**Table 1:** Combination of key hyperparameters.

Configuration	Epochs	Learning Rate	ReducedLRonPlateau
Config-1	200	0.0005	0.2
Config-2	200	0.0005	0.02
Config-3	100	0.00005	0.2

### 4.2 Training

Neural networks function similarly to how people acquire knowledge: first, the computer is instructed to understand a set of concepts; then, using these, it can extrapolate responses to unforeseen circumstances. During this learning phase, a process of developing a relationship between the data inputs and the intended outputs takes place behind the scenes. Investigating the relationship between an image and its caption is crucial to successfully applying this mapping to the task at hand. To determine which combination (CNN and RNN) produces the best results, several well-known deep learning models have been trained on the updated dataset to achieve accuracy comparable to the desired output. An attention mechanism is introduced into a setup comprising nine different models, including three distinct CNN models (VGG19, ResNet50v2, and Inception v3) and three distinct RNN models (LSTM, GRU, and BiLSTM). When it comes to training, one of the most essential factors to consider is the number of epochs, as having too many or too few can lead to overfitting or underfitting the model. This issue can be addressed by using a tactic known as early stopping, also called a callback approach. This technique allows for creating an indefinitely large number of epochs but will automatically halt training if there is no longer any performance improvement.

## 5 Result analysis

The results of the recommended models on the testing dataset are displayed in Table 2, along with the criteria used to select the best alternative model. The quantitative analysis of image captioning in the Bengali language provides significant insights into the effectiveness of the proposed methodology. Each combination of CNN and RNN models is systematically evaluated based on optimal hyperparameter settings. Subsequently, captions are generated for each model using two distinct search strategies. To facilitate the creation of Bengali captions, the decoding process involves exploring all potential output sequences to determine the most likely ones. The proposed method integrates beam search and greedy search strategies to generate captions that accurately describe the images. This approach ensures a comprehensive exploration of possible output sequences while optimizing the efficiency of the captioning process.

**Table 2:** Performance of various CNN and attention-based RNN combinations.

CNN	Attention + RNN	Accuracy	Value	Loss	Value
VGG19	LSTM	0.7701	0.7059	0.7917	1.3311
ResNet50v2		0.7591	0.6952	1.0352	1.4753
Inceptionv3		0.7479	0.7009	1.0637	1.4216
VGG19	BiLSTM	0.7951	0.7064	0.6739	1.3583
ResNet50v2		0.7814	0.7066	0.8825	1.3970
Inceptionv3		0.8066	0.7171	0.7078	1.3483
VGG19	GRU	0.8005	0.7192	0.6685	1.2633
ResNet50v2		0.7315	0.6912	1.1860	1.5121
Inceptionv3		0.7691	0.7142	0.9628	1.3668

### 5.1 Performance of greedy search

Greedy search for image captioning is efficient and effective in generating descriptive captions. This method works by selecting the most probable word at each decoding step based on the highest probability predicted by the model. While this approach simplifies the decoding process and requires less computational power than more complex search algorithms, it may produce sub-optimal results by choosing locally optimal words at each step. Despite this limitation, greedy search often generates fluent and coherent captions quickly, making it suitable for real-time applications where speed is crucial. Its performance is awe-inspiring when used with models that produce well-calibrated probability distributions. The balance between computational efficiency and linguistic fluency makes greedy search a valuable technique for image captioning. Table 3 provides detailed insights into these aspects.

**Table 3:** Greedy search performance analysis for different CNN and RNN configurations.

CNN	RNN	BLEU1	BLEU2	BLEU3	BLEU4	METEOR	ROUGE
VGG19	LSTM	0.685	0.568	0.431	0.387	0.337	0.423
ResNet50v2		0.363	0.339	0.287	0.240	0.167	0.334
Inceptionv3		0.408	0.321	0.300	0.285	0.197	0.363
VGG19	BiLSTM	0.337	0.302	0.253	0.185	0.128	0.356
ResNet50v2		0.500	0.408	0.340	0.320	0.220	0.38
Inceptionv3		0.544	0.433	0.387	0.309	0.215	0.356
VGG19	GRU	0.699	0.614	0.524	0.341	0.314	0.394
ResNet50v2		0.487	0.432	0.336	0.284	0.197	0.330
Inceptionv3		0.418	0.375	0.305	0.244	0.169	0.370
VGG19	LSTM + Attention	0.629	0.572	0.488	0.407	0.317	0.467
ResNet50v2		0.418	0.377	0.313	0.299	0.207	0.366
Inceptionv3		0.537	0.482	0.391	0.362	0.251	0.356
VGG19	BiLSTM + Attention	0.295	0.260	0.207	0.158	0.109	0.327
ResNet50v2		0.513	0.465	0.397	0.392	0.272	0.359
Inceptionv3		0.576	0.522	0.456	0.422	0.293	0.369
VGG19	GRU + Attention	0.681	0.585	0.492	0.422	0.337	0.489
ResNet50v2		0.502	0.460	0.387	0.309	0.214	0.350
Inceptionv3		0.571	0.522	0.448	0.430	0.298	0.366

### 5.2 Performance of beam search

The beam search method uses a heuristic approach to select the top K decoding solutions based on the conditional probabilities at each node in the input sequence. In this experiment, a beam width of k = 3 is used, which means that three potential options are considered at each step before making a final decision. The search process for each sequence concludes when it reaches a maximum length of 10 words, an end-of-sequence token, or a specific threshold probability. This method benefits from normalizing sentence length, which contributes to generating higher-quality output sentences. Table 4 shows the BLEU, METEOR, and ROUGE scores for the beam search approach, both with and without the attention mechanism.

**Table 4:** Beam search performance analysis for different CNN and RNN configurations.

CNN	RNN	BLEU1	BLEU2	BLEU3	BLEU4	METEOR	ROUGE
VGG19	LSTM	0.645	0.585	0.451	0.390	0.330	0.433
ResNet50v2		0.376	0.335	0.265	0.263	0.174	0.315
Inceptionv3		0.568	0.507	0.408	0.337	0.224	0.355
VGG19	BiLSTM	0.307	0.282	0.230	0.195	0.134	0.334
ResNet50v2		0.415	0.390	0.360	0.305	0.220	0.349
Inceptionv3		0.490	0.436	0.405	0.326	0.250	0.334
VGG19	GRU	0.620	0.558	0.472	0.346	0.226	0.396
ResNet50v2		0.585	0.510	0.401	0.299	0.199	0.344
Inceptionv3		0.418	0.372	0.314	0.304	0.202	0.387
VGG19	LSTM + Attention	0.708	0.578	0.505	0.432	0.389	0.470
ResNet50v2		0.565	0.498	0.414	0.390	0.259	0.323
Inceptionv3		0.656	0.590	0.485	0.395	0.295	0.334
VGG19	BiLSTM + Attention	0.323	0.286	0.230	0.172	0.114	0.309
ResNet50v2		0.598	0.542	0.405	0.400	0.225	0.355
Inceptionv3		0.672	0.602	0.510	0.408	0.280	0.435
VGG19	GRU + Attention	0.690	0.605	0.499	0.425	0.358	0.465
ResNet50v2		0.624	0.562	0.453	0.327	0.217	0.327
Inceptionv3		0.627	0.545	0.432	0.350	0.278	0.418

### 5.3 SHAP analysis

In image captioning, SHAP (Shapley Additive Explanations) analysis is employed to identify which regions of an image contribute most to the generation of specific words or phrases in the caption [31]. This research, which focuses on generating descriptive captions in Bengali using visual attention mechanisms, integrates SHAP analysis to understand these contributions better. We use GradientExplainer, a variant of SHAP tailored for deep learning models, which is especially effective with convolutional neural networks and transformers. GradientExplainer computes SHAP values by evaluating the gradients of outputs concerning inputs, facilitating efficient calculation of these values for high-dimensional data like images. For each generated caption, SHAP values are calculated to pinpoint which areas of the image most significantly influence specific words in the caption. This process involves modifying the standard input-output relationship as follows:

$$\begin{array}{c} \varphi_{i}=\sum_{S\subseteq F\backslash \{i\}}\frac{|S|!(|F|-|S|-1)!}{|F|!}\left[f\left(S\cup \left\{i\right\}\right)-f\left(S\right)\right] \end{array}$$ (1)

where *ϕ*_*i*_ is the SHAP value for pixel *i*, *F* is the set of all pixels, *S* is a subset of pixels excluding *i*, and *f* is the model output function.

**Fig 3:**
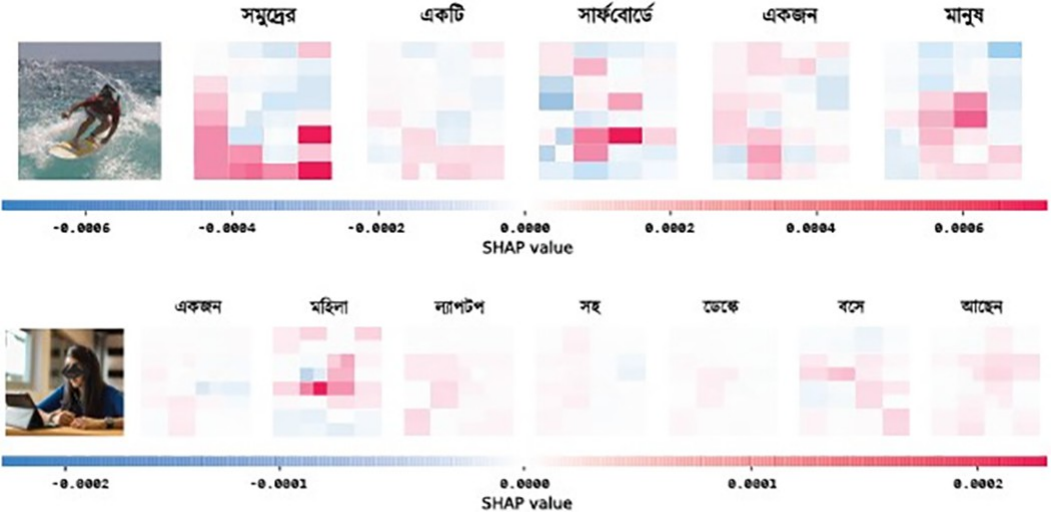
SHAP value heatmap for a sample image and its corresponding Bangla caption. Positive SHAP values (in red) highlight regions contributing positively towards the caption prediction, while negative values (in blue) indicate inhibitory regions.

The experimental setup for this analysis involves processing images through the visual transformer and then computing the SHAP values for each component of the generated caption. The resulting SHAP values are visualized as heatmaps overlaid on the original images, showing the pixel-level contributions to the caption. Fig 3 shows an example of one such heatmap.

### 5.4 Qualitative analysis

**Fig 4:**
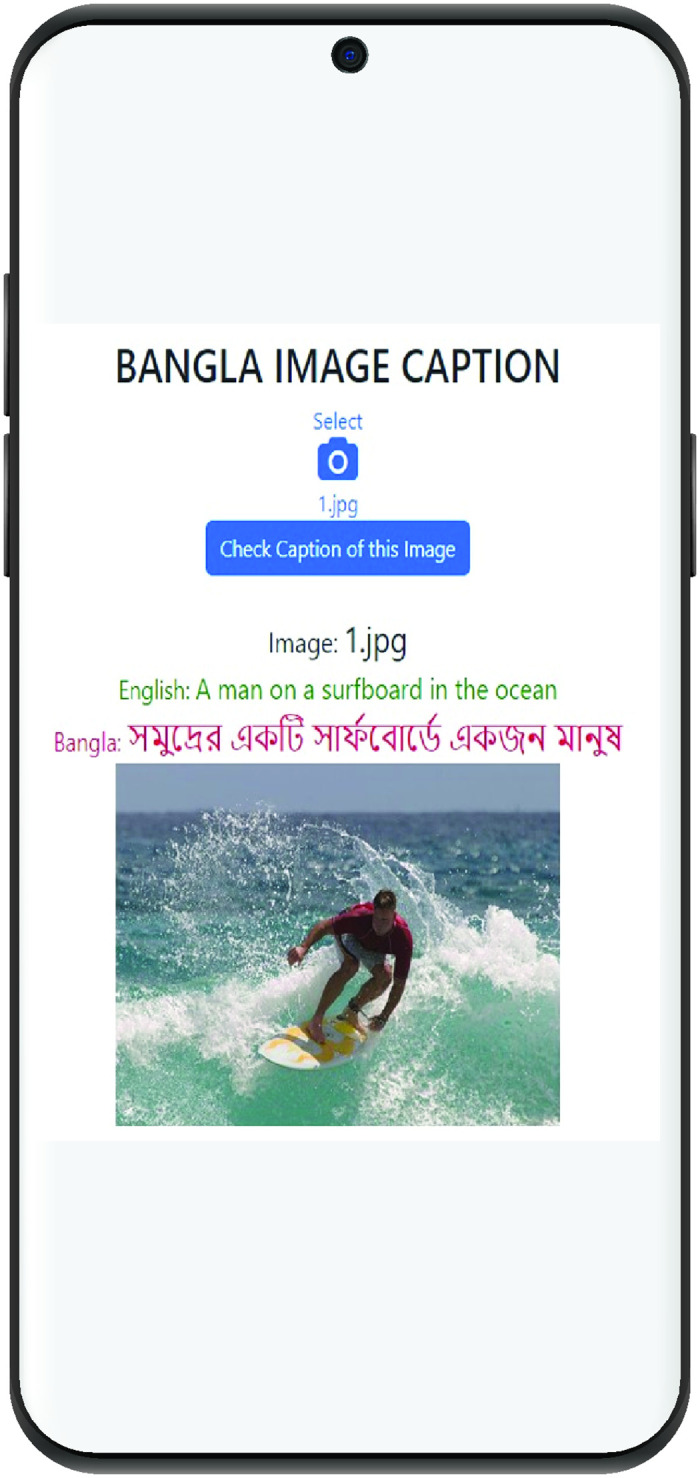
Developed application for Bangla image captioning.

This section illustrates the practical implementation of the proposed model, showcasing its output. The predicted Bengali caption, displayed in Fig 4, closely matches the reference caption, which is shown in English.

### 5.5 Performance comparison

A comparison is made between the proposed Bengali image captioning model and existing models. Bengali has been relatively underrepresented in image captioning, leading to limited research in this area. The results of the proposed model are evaluated against previous research on Bengali image captioning. Table 5 compares the performance metrics of various models, contrasting them with the results of the proposed model.

**Table 5:** Performance comparison of suggested and existing models.

Model	BLEU3	BLEU4	METEOR	ROUGE
Proposed Model	0.451	0.390	0.330	0.433
Proposed Model + Attention	**0.505**	**0.432**	**0.389**	**0.470**
Humaira et al. [32]	0.454	0.344	-	-
Kamal et al. [33]	0.315	0.238	0.182	-

## 6 Conclusion

The automated generation of image captions in the complex Bengali language poses a significant challenge in artificial intelligence. Integrating computer vision and natural language processing algorithms adds to this complexity, especially given the limited availability of Bengali-captioned image datasets. This study addresses these challenges by introducing a carefully human-annotated dataset of Bengali captions and employing an innovative end-to-end architecture with an attention-driven decoder for generating relevant image descriptions in Bengali. By using Gated Recurrent Units (GRUs) to capture spatial and temporal attributes and incorporating an attention mechanism to explore the relationship between visual and linguistic representations, the model lays the groundwork for constructing coherent sentences. The model’s recursive unit, consisting of two layers, leverages these attention mechanisms to achieve notable performance, with training results showing a BLEU-4 score of 43%, a METEOR score of 39%, and a ROUGE score of 47%. These results surpass previous efforts in Bengali image captioning, highlighting the model’s leading position in the field.

However, the main challenge of this research lies in generating context-aware Bengali language captions due to the limitations of the dataset and the specific cultural nuances of the Bengali language. The model may struggle with particularly complex Bengali sentence structures or nuanced cultural references within images. Additionally, existing evaluation metrics (BLEU-4, METEOR, ROUGE) may not fully capture the subtleties of natural Bengali captions. Future work could focus on developing evaluation metrics that assess fluency, grammatical accuracy, and cultural relevance specific to Bengali. Furthermore, exploring alternative architectures, such as transformers with pre-trained Bengali language models, could improve the model’s handling of complex sentence structures. Domain-specific or context-aware training for news images, historical photographs, or artistic creations could also enhance caption accuracy in these domains.
